# Effects of percutaneous kyphoplasty with concomitant postural reduction in the treatment of acute osteoporotic vertebral compression fractures

**DOI:** 10.3389/fmed.2026.1969002

**Published:** 2026-09-28

**Authors:** Yulin Chen, Peng Yang, Ankejiang Anniwaer, Zhengyu Xu, Huilin Yang, Quan Zhou, Tao Liu

**Affiliations:** Department of Orthopaedics, The First Affiliated Hospital of Soochow University, Soochow University, Suzhou, China

**Keywords:** anterior vertebral height, lumbar lordosis, OVCF, PKP, postural reduction

## Abstract

**Objective:**

To investigate the effects of postural reduction during balloon expansion in percutaneous kyphoplasty (PKP) for patients with acute osteoporotic vertebral compression fractures (OVCF).

**Methods:**

From January 2022 to December 2023, 75 patients with acute OVCF underwent PKP surgery at our institution and were followed up for at least 12 months. Patients were divided into Group A and Group B based on whether they underwent postural reduction during balloon expansion. Clinical evaluation included Oswestry Disability Index (ODI) and Visual Analog Scale (VAS) scores for back pain; Radiographic evaluation included lumbar lordosis angle (LL), anterior vertebral height (AVH) and rate (AVHr).

**Results:**

There were no significant differences in baseline clinical and radiographic parameters between the two groups (*P* > 0.05). Both groups showed significant improvement in VAS, ODI, and LL at one day, one month, and final follow-up post-surgery compared to preoperative levels (*P* < 0.05). Group A exhibited significantly greater improvements in ODI, LL, AVH, and AVHr at the final follow-up compared to Group B (*P* < 0.05). Regarding complications, including cement leakage, adjacent segment fractures and refracture of the treated vertebral body, there was no statistically significant difference between Group A and Group B (*P* > 0.05). No significant between-group differences were observed in the recorded complications. Patients who developed adjacent vertebral fractures or treated-vertebra recollapse were managed nonoperatively, and none required reoperation during follow-up.

**Conclusion:**

PKP combined with postural reduction during balloon expansion is an effective treatment for acute OVCF, as it can further restore vertebral height, and regional lumbar alignment. Hence, it is recommended for clinical application in suitable patients.

## Introduction

As the global population ages, osteoporosis is becoming increasingly prevalent, placing a substantial burden on healthcare systems worldwide. Osteoporotic vertebral compression fractures (OVCF), one of the most common complications of osteoporosis, severely impact the physical and mental health of the elderly population in China (1–3). Clinically, OVCF often present as back pain, height loss, kyphosis, and even reduced pulmonary function. In severe cases, patients may lose the ability to work or live independently, posing a significant threat to their health and quality of life (4, 5). Conservative treatment has traditionally been ineffective in rapidly relieving pain or restoring vertebral height. Patients typically require prolonged bed rest, which increases the risk of complications such as pulmonary infections, pressure ulcers, and urinary tract infections (6–10). Therefore, the primary goals of OVCF treatment are pain relief and deformity correction to enable early mobilization, reducing complications and the occurrence of chronic pain (11).

Percutaneous kyphoplasty (PKP), first introduced by Mark Reiley et al. in 1999, is a minimally invasive surgical technique for treating OVCF (12). During the procedure, a small incision is made in the patient's back, and a specialized trocar is guided into the vertebra under fluoroscopy. A balloon is then inserted to restore the compressed vertebral body, followed by the injection of bone cement to stabilize the vertebra (13, 14). PKP significantly alleviates pain, restores vertebral height, and corrects kyphotic deformities (15–18).

However, despite its advantages, PKP may leave residual kyphotic deformities, which could affect long-term outcomes (19–21), particularly in patients with severe osteoporosis (22). This limitation may arise because osteoporotic bone cannot withstand the high pressures required for adequate balloon expansion, leading to limited restoration of vertebral height and insufficient correction of kyphotic deformities. Postural reduction may facilitate partial restoration of vertebral height in acute OVCF with residual fracture mobility. Ligamentotaxis refers to indirect fracture reduction achieved by applying tension through the intact soft-tissue structures surrounding a fracture. In OVCF, this mechanism differs from its conventional application in high-energy traumatic burst fractures, in which longitudinal distraction is used to reduce displaced or retropulsed fracture fragments. When the posterior vertebral wall and ligamentous structures remain intact, prone extension and gentle external reduction may tension the anterior longitudinal ligament and adjacent annular structures, creating an extension moment across the mobile compressed vertebra and permitting partial re-expansion of the anterior column (23). Because osteoporotic bone is mechanically fragile, this maneuver should be performed gently under fluoroscopic guidance and should not be extrapolated to unstable burst fractures, posterior wall disruption, ligamentous injury, or neurological compromise. Since OVCF rarely result in burst fractures extending through the axial plane, postural reduction is generally safe for these patients (24, 25). By partially restoring vertebral height, postural reduction increases the volume of the compressed vertebra, facilitates trocar placement, and enhances balloon expansion (26).

Previous studies have primarily evaluated postural reduction performed before vertebroplasty or kyphoplasty as an adjunctive positioning maneuver. However, the additional effect of applying manual postural reduction concurrently with balloon inflation during PKP has not been adequately investigated. This intraoperative approach differs from preoperative postural reduction because external manual force and balloon expansion are applied simultaneously and may act together to restore vertebral height. Nevertheless, it remains unclear whether this combined maneuver provides additional radiographic or clinical benefits over PKP alone without increasing procedure-related complications. Therefore, we conducted a retrospective comparative cohort study to compare PKP with intraoperative postural reduction during balloon inflation versus PKP alone in patients with acute thoracolumbar OVCF. We evaluated pain, functional outcomes, anterior vertebral height restoration, lumbar lordosis, and complications. We hypothesized that intraoperative postural reduction would improve vertebral height restoration while maintaining clinical outcomes and complication rates comparable to those of PKP alone.

## Methods

### Inclusion criteria

Patients meeting the following criteria were included: (1) Single-level thoracolumbar vertebral compression fracture (T11-L3); (2) Osteoporosis confirmed by dual-energy x-ray absorptiometry (DEXA) with a T-score ≤ −2.5; (3) Vertebral anterior height (AVH) reduction ≥ 30%(The estimated prefracture anterior height was calculated as the mean anterior height of the adjacent nonfractured vertebrae above and below the index level.), consistent with the diagnosis of OVCF; (4) Significant back pain without neurological symptoms; (5) Patients undergoing PKP; (6) Fractures diagnosed within two weeks of onset.

### Exclusion criteria

The following patients were excluded: (1) History of spinal fractures or surgical interventions; (2) Fractures associated with tumors, tuberculosis, or ankylosing spondylitis; (3) Inability to complete follow-up due to other factors.

### General information

According to the inclusion and exclusion criteria, a total of 75 patients with acute OVCF were enrolled from January 2022 to December 2023, including 31 treated with PKP combined with postural reduction during balloon expansion (Group A) and 44 treated with PKP alone (Group B) (Figure 1). All procedures were performed by the same surgical team. Treatment selection was not randomized. The decision to perform postural reduction during balloon inflation was made after evaluation of the preoperative imaging findings and discussion of the potential benefits and risks with the patient. The patients' data and imaging results come from the hospital's electronic medical records. Follow-up evaluations were conducted one day, one month, and one year after surgery, with a minimum follow-up period of 12 months (Figure 2).

**Figure 1:**
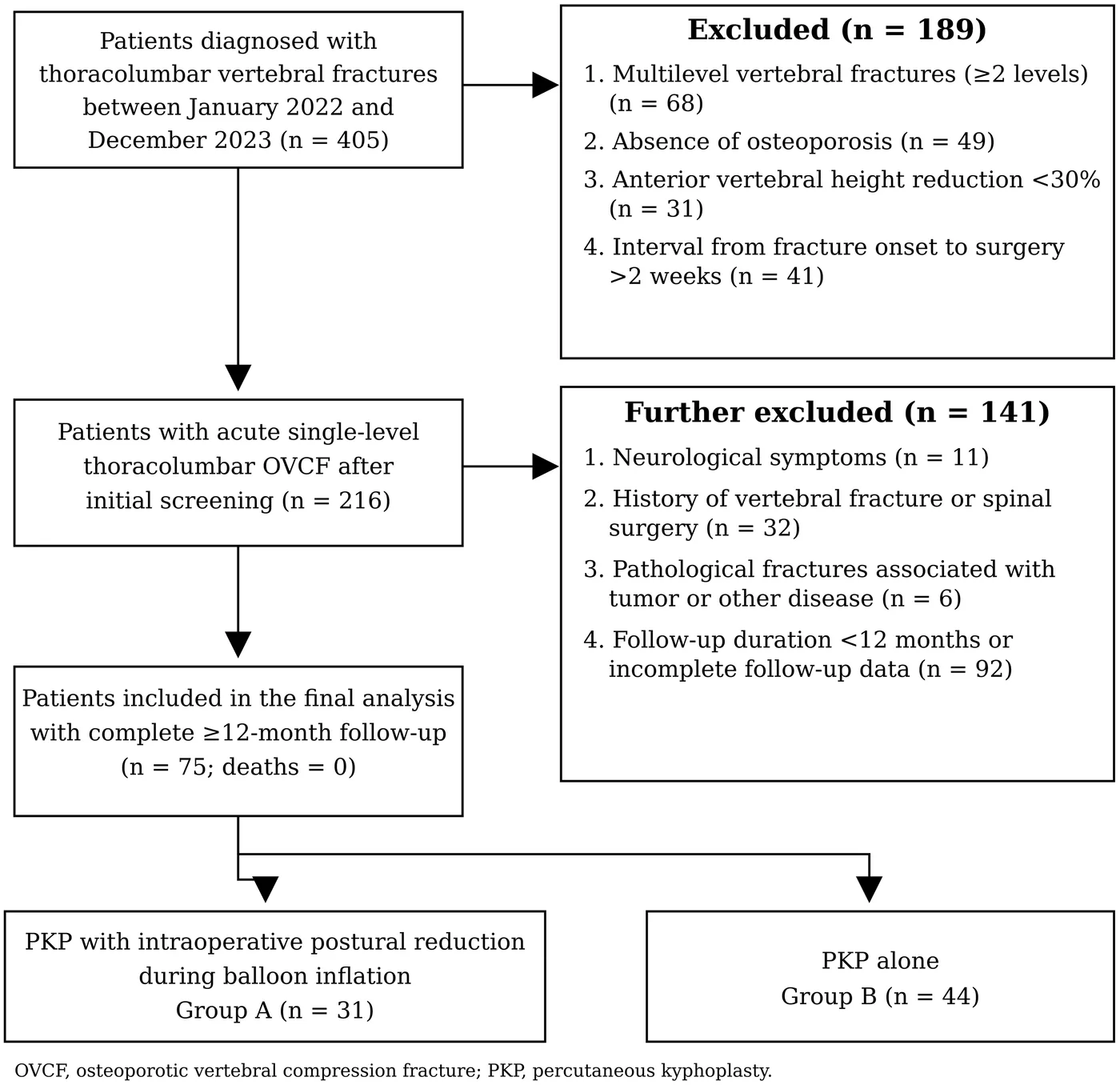
Patient selection flowchart for inclusion in this study.

**Figure 2:**
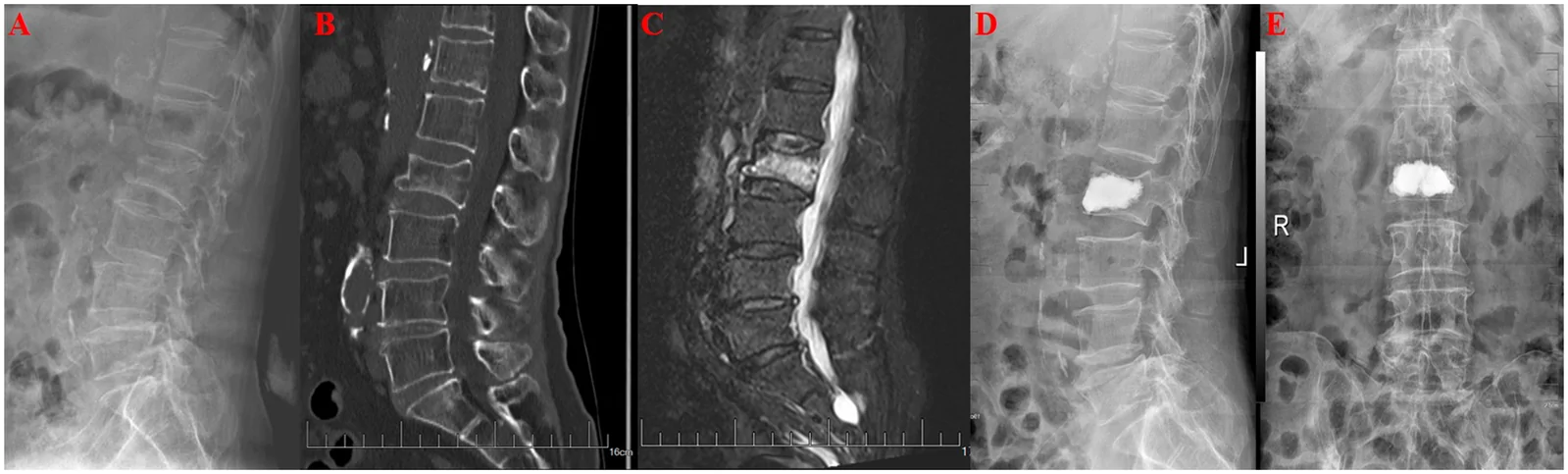
Preoperative and postoperative images: **(A)** preoperative lateral radiograph of the thoracolumbar spine; **(B)** preoperative sagittal CT image; **(C)** preoperative sagittal MRI image; **(D)** postoperative lateral radiograph; and **(E)** postoperative anteroposterior radiograph.

### Surgical procedure

#### Group A

Patients were positioned prone on the operating table with the abdomen suspended. Under fluoroscopic guidance, the fractured vertebra was marked. C-arm fluoroscopy was used to confirm parallel endplates on the lateral view and symmetric pedicles on the anteroposterior view. Following general anesthesia, a 5-mm incision was made. A trocar was inserted into the vertebral pedicle under fluoroscopic guidance, with the needle tip reaching the posterior margin of the vertebral body in the lateral view and the medial edge of the pedicle in the anteroposterior view. A guidewire was introduced, and the trocar was replaced with a working cannula to create a channel using a hand drill. After establishment of the working channel, the balloon was inserted into the fractured vertebral body and gradually inflated. Simultaneously, the same surgeon placed both palms, one over the other, directly over the spinous process corresponding to the fractured vertebra and applied a gradual posterior-to-anterior force, approximately perpendicular to the operating table. Manual pressure was initiated at the beginning of balloon inflation and was maintained continuously until balloon inflation was completed. The maneuver was discontinued when satisfactory vertebral height restoration was achieved, no further reduction was observed, cortical contact was suspected, or the predetermined maximum balloon pressure was reached.

Balloon position, vertebral height, and cortical contour were evaluated using intermittent C-arm fluoroscopy throughout balloon inflation and manual reduction. Intraoperative neurophysiological monitoring was used during the reduction maneuver to detect potential neurological compromise. All manual reduction procedures were performed by the same surgeon using the same hand position, direction of force, and operative sequence. The magnitude of the externally applied force was not measured using a force-sensing device. Instead, the force was increased gradually and adjusted according to the surgeon's tactile feedback, fluoroscopic findings, and neurophysiological monitoring. The duration of manual pressure corresponded to the balloon-inflation period and was not recorded separately (Figure 3).

**Figure 3:**
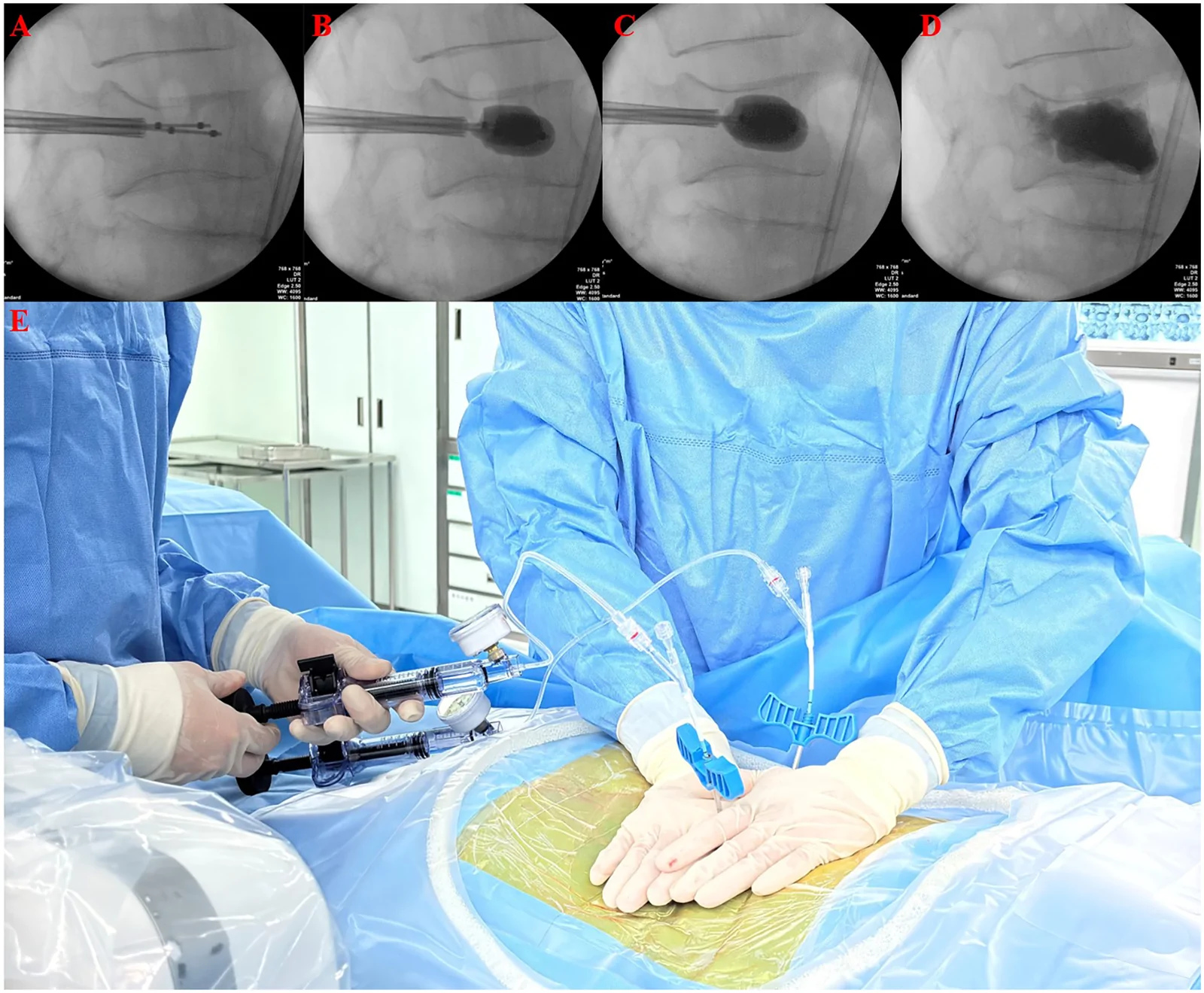
Surgical procedure: **(A)**: balloon insertion; **(B)**: completion of balloon expansion; **(C)**: balloon expansion combined with postural reduction; **(D)**: completion of bone cement injection; **(E)**: balloon expansion combined with postural reduction during surgery.

#### Group B

Patients in Group B underwent the same anesthesia, prone positioning, operative preparation, balloon-inflation protocol, intermittent C-arm fluoroscopic monitoring, neurophysiological monitoring, and cement-injection procedure as patients in Group A. Passive reduction resulting from prone positioning and balloon inflation could therefore occur in both groups. The only procedural difference was that no manual external pressure was applied to the thoracolumbar region during balloon inflation in Group B.

Postoperative care included anti-osteoporosis therapy and limited ambulation with lumbar support within 24 h. Patients were advised to perform back muscle strengthening exercises and continue anti-osteoporosis medications during follow-up.

### Clinical evaluation

Patients were assessed preoperatively, at one day, one month, and one year postoperatively using the following tools: (1) **Oswestry Disability Index (ODI):** Evaluates improvements in patient quality of life. (2) **Visual Analog Scale (VAS):** Measures subjective pain intensity on a scale of 0–10, with 0 representing no pain and 10 indicating the worst possible pain.

VAS and ODI scores were obtained from the routine clinical assessments documented at the preoperative evaluation and at 1 day, 1 month, and 1 year postoperatively. The assessments were extracted and recorded by a dedicated research staff member who was not involved in the surgical procedures. Because of the retrospective nature of the study, formal blinding during the original clinical assessments could not be guaranteed.

### Radiographic evaluation

(1) **Bone Mineral Density (BMD):** Measured using dual-energy x-ray absorptiometry (DEXA; Discovery Wi, Hologic, USA), with T-scores recorded (27); (2) **Anterior Vertebral Height (AVH):** Calculated as H_f_/H_ref_   ×   100%, where H_f_ was the anterior height of the fractured vertebra and H_ref_ was the mean anterior height of the adjacent nonfractured vertebrae above and below the index level. Measured from lateral x-ray images preoperatively and at one day, one month, and one year postoperatively; (3) **Anterior Vertebral Height Restoration Rate (AVHr):** Calculated as the ratio of restored height to the pre-fracture height; (4) **Lumbar Lordosis (LL):** Defined as the angle between the superior endplate of L1 and the superior endplate of S1 (Figure 4).

**Figure 4:**
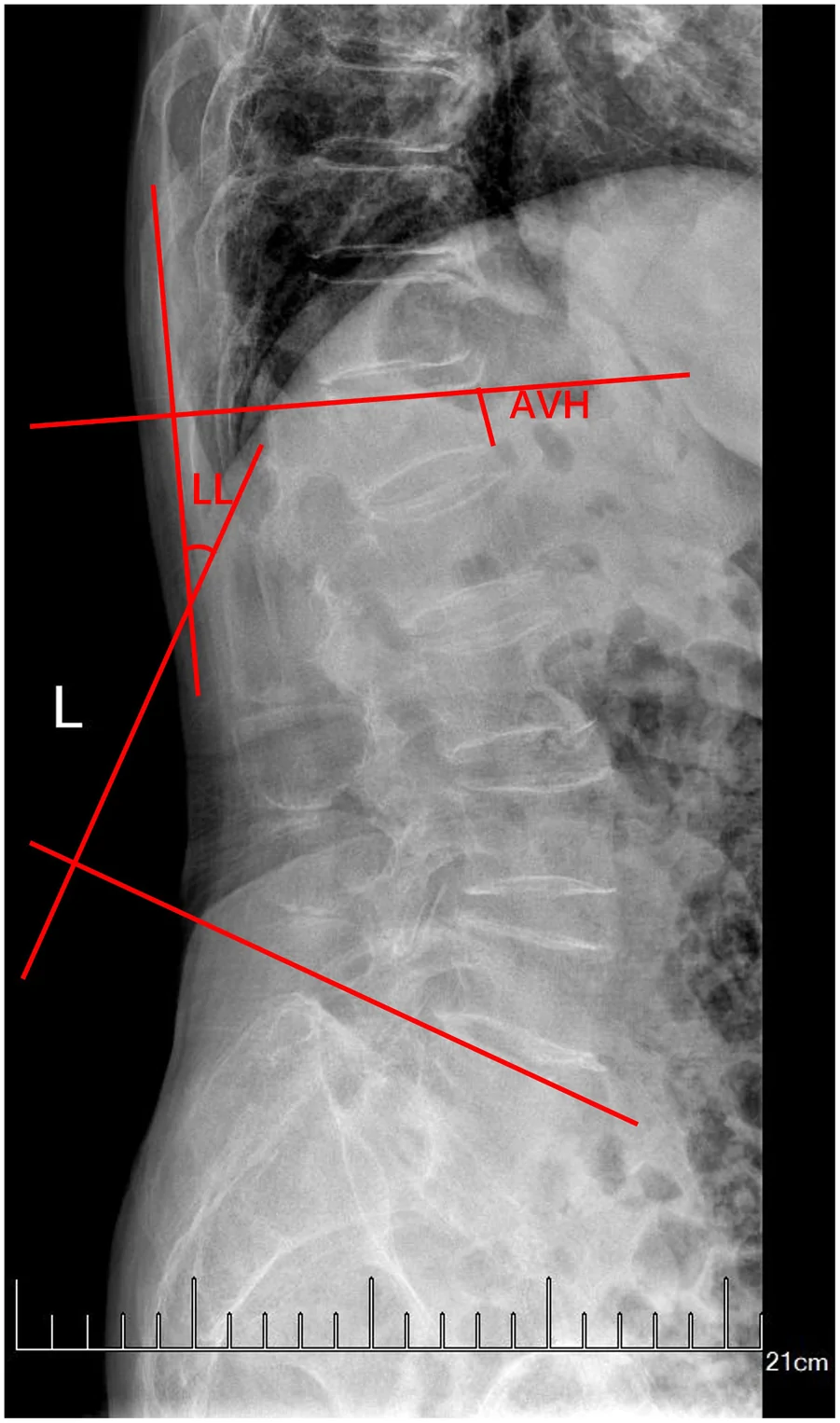
Plain lateral radiographs for measuring pre-operative parameters. LL: Lumbar lordosis; AVH: Anterior vertebral height.

Radiographic measurements were performed using the digital measurement tools available on the hospital picture archiving and communication system (PACS) workstation. All radiographic parameters were measured by a trained observer who was not blinded to treatment allocation or clinical outcomes. The measurements obtained by this observer were used in the statistical analysis.

Additionally, complications such as cement leakage, adjacent segment fractures, and vertebral collapse were documented during follow-up.

### Statistical analysis

Data were processed using SPSS 26.0 software (SPSS Inc., Chicago, IL). Continuous variables were expressed as mean ± standard deviation (x̄ ± s) and compared within groups using paired t-tests. Categorical variables were analyzed using chi-square tests. A *P*-value < 0.05 was considered statistically significant.

## Results

### Baseline characteristics

The demographic and clinical characteristics of the patients are summarized in Table 1. The mean age of the cohort was 70 years, with fewer male patients (20 cases) than female patients (55 cases). The distribution of fractured vertebral levels is presented in Table 1. L1 was the most frequently affected vertebra in the overall cohort, accounting for 24 of 75 cases (32.00%). L1 was also the most commonly affected level in both Group A (32.26%) and Group B (31.82%). There were no statistically significant differences between Groups A and B in age, sex, trauma history, or fracture location (*P* > 0.05). The mean body mass index (BMI) of Group A (25.19) was slightly higher than that of Group B (25.11), but this difference was not statistically significant (*P* > 0.05). The mean BMD T-score for all patients was −2.86, with no significant differences between the groups (*P* > 0.05). Similarly, the prevalence of comorbidities such as hypertension, diabetes, hyperlipidemia, and smoking did not differ significantly between the groups (*P* > 0.05). The mean follow-up duration was 17.45 ± 6.60 months, with no significant difference between Group A and Group B (17.29 ± 6.83 vs. 17.57 ± 6.51 months), with no significant differences between groups (*P* > 0.05).

**Table 1:** General characteristics of the patients.

Characteristic	Full sample	Group A	Group B	*P-*Value
Number of patients	75	31	44	
Age (years)	70.15 ± 6.37	69.68 ± 6.02	70.48 ± 6.66	0. 596
Gender (male/female)	20/55	9/22	11/33	0.697
Fractured segment (n)				0.833
T11	16 (21.33%)	5 (16.13%)	11 (25%)	
T12	11 (14.67%)	5 (16.13%)	6 (13.64%)	
L1	24 (32.00%)	10 (32.26%)	14 (31.82%)	
L2	18 (24.00%)	9 (29.03%)	9 (20.45%)	
L3	6 (8.00%)	2 (6.45%)	4 (9.09%)	
BMI (kg/m^2^)	25.14 ± 3.64	25.19 ± 3.75	25.11 ± 3.60	0. 921
BMD (T-score)	−2.86 ± 0.53	−2.85 ± 0.60	−2.87 ± 0.48	0. 889
Comorbidity (n)				
Hypertension	26 (34.67%)	12 (38.71%)	14 (31.82%)	0.537
Diabetes	22 (29.33%)	9 (29.03%)	13 (29.55%)	0.962
Hyperlipidemia	25 (33.33%)	11 (35.48%)	14 (31.82%)	0.740
Smoking	21 (28.00%)	9 (29.03%)	12 (27.27%)	0.867
Alcoholism	17 (22.67%)	6 (19.35%)	11 (25.00%)	0.565
Follow-up (months)	17.45 ± 6.60	17.29 ± 6.83	17.57 ± 6.51	0.859

The *p*-values for the fracture segment distribution represents the comparison of the full distribution.

BMI, body mass index, BMD, bone mineral density.

### Clinical outcomes

Both groups demonstrated significant reductions in VAS and ODI scores at all postoperative time points compared with their respective preoperative values (all *P* < 0.05). No statistically significant between-group difference in VAS score was detected at postoperative day 1, 1 month, or 1 year (all *P* > 0.05), indicating that no additional analgesic benefit associated with intraoperative postural reduction was detected.

No statistically significant difference in ODI score was observed between the groups at postoperative day 1 or 1 month. At the 1-year follow-up, the ODI score was statistically lower in Group A than in Group B (18.71 ± 4.72 vs. 21.55 ± 4.92, nominal *P* = 0.015), corresponding to an absolute between-group difference of 2.84 points. However, this difference was below the commonly reported MCID of approximately 10–12.8 points for the ODI (Table 2).

**Table 2:** Comparison of functional outcomes.

Time point	Group A (*n* = 31)	Group B (*n* = 44)	*P*-Value
VAS			
Preop	7.65 ± 0.98	7.70 ± 0.70	0.761
Postop 1d	3.42 ± 0.85*	3.36 ± 0.78*	0.770
Postop 1m	2.00 ± 1.00*	1.90 ± 1.03*	0.704
Postop 1y	2.16 ± 0.73*	2.27 ± 1.02*	0.604
ODI			
Preop	55.55 ± 9.77	56.57 ± 10.84	0.677
Postop 1d	25.13 ± 2.86*	25.14 ± 2.59*	0.991
Postop 1m	17.61 ± 5.49*	17.89 ± 5.36*	0.830
Postop 1y	18.71 ± 4.72*	21.55 ± 4.92*	**0.015** **

ODI: oswestry disability index; VAS: visual analogue scale.

Bold *P* values indicate statistically significant between-group differences (*P* < 0.05).

*Significance compared with the preoperative, *P* < 0.05.

**Significance between the two groups, *P* < 0.05.

### Radiographic outcomes

Both groups demonstrated significant improvements in AVH and AVHr at one day, one month, and one year postoperatively compared to preoperative measurements (*P* < 0.05). Group A achieved significantly better AVH and AVHr recovery at all postoperative time points compared to Group B (*P* < 0.05). Both groups experienced significant increases in LL postoperatively (*P* < 0.05). While there was no significant difference in LL recovery between the groups at one day or one month postoperatively (*P* > 0.05), At the 1-year follow-up, the AVH percentage remained higher in Group A than in Group B (64.03 ± 7.17% vs. 59.92 ± 8.29%), corresponding to an absolute between-group difference of 4.11 percentage points. Group A also had a greater LL angle than Group B at 1 year (42.68 ± 4.56° vs. 39.59 ± 6.40°), with an absolute difference of 3.09°. (*P* < 0.05) (Table 3).

**Table 3:** Comparison of radiographic parameters.

Time point	Group A (*n* = 31)	Group B (*n* = 44)	*P*-Value
AVH (%)			
Preop	50.65 ± 7.51	50.68 ± 7.87	0.989
Postop 1d	68.88 ± 8.53*	63.29 ± 8.37*	**0.006** **
Postop 1m	67.57 ± 8.65*	61.62 ± 8.16*	**0.003** **
Postop 1y	64.03 ± 7.17*	59.92 ± 8.29*	**0.028** **
AVHr (%)			
Postop 1d	18.22 ± 6.94	12.62 ± 5.70	**<0.001** **
Postop 1m	16.92 ± 7.17	10.95 ± 5.76	**<0.001** **
Postop 1y	13.38 ± 6.44	9.24 ± 6.00	**0.006** **
Lumbar lordosis (°)			
Preop	35.35 ± 4.74	35.39 ± 6.52	0.982
Postop 1d	46.03 ± 5.21*	43.86 ± 6.85*	0.142
Postop 1m	44.65 ± 5.02*	42.32 ± 6.44*	0.097
Postop 1y	42.68 ± 4.56*	39.59 ± 6.40*	**0.024** **

AVH, anterior vertebral height; AVHr, anterior vertebral height recovery rate.

(postoperative AVH/pre-fracture height).

Bold *P* values indicate statistically significant between-group differences (*P* < 0.05).

*Significance compared with the preoperative, *P* < 0.05.

**Significance between the two groups, *P* < 0.05.

### Complications

Bone cement leakage occurred in 3 patients in Group A (9.68%) and 2 patients in Group B (4.55%). Although the leakage rate was numerically higher in Group A, the absolute difference of 5.13 percentage points was not statistically significant (*P* = 0.643). All five cases of cement leakage were asymptomatic, with no neurological deficits, pulmonary symptoms, or requirement for additional intervention. Adjacent segment fractures occurred in 1 case in Group A and 2 cases in Group B, with no significant difference between the groups (*P* > 0.05). Vertebral collapse was observed in 1 patient in Group B but was not observed in Group A. However, this difference was not statistically significant (*P* > 0.05). These patients received nonoperative management, including analgesic treatment, bracing, activity modification, and continued anti-osteoporosis therapy, as clinically indicated. No new neurological deficits, persistent severe pain, or marked deterioration in activities of daily living was documented during subsequent follow-up, and none of these patients underwent additional surgery (Table 4).

**Table 4:** Bone cement characteristics and complications.

Parameter	Group A (*n* = 31)	Group B (*n* = 44)	*P*-Value
Bone cement volume (mL)	7.42 ± 1.12	7.18 ± 1.02	0.343
Overall cement leakage (n)	3 (9.68%)	2 (4.55%)	0.643
Intradiscal leakage (n)	1 (3.23%)	1 (2.27%)	
Paravertebral leakage (n)	2 (6.45%)	1 (2.27%)	
Intraspinal leakage (n)	0 (0%)	0 (0%)	
Adjacent vertebral fracture (n)	1 (3.23%)	2 (4.55%)	1.000
Treated vertebral recollapse (n)	0 (0%)	1 (2.27%)	1.000

All cement leakages were asymptomatic and required no additional treatment. *P* values for complications were calculated using Fisher's exact test; cement leakage subtypes were summarised descriptively and were not tested separately.

## Discussion

Previous studies have evaluated postural reduction mainly as a preprocedural maneuver before vertebral augmentation. Chin et al. (28) performed postural reduction with a pillow placed beneath the fractured vertebra before PVP and reported improved vertebral height and Cobb angle. Yang et al. (29) compared hyperextension postural reduction before vertebroplasty with *in situ* vertebroplasty and observed greater improvement in anterior vertebral height, particularly in patients with severe vertebral compression. In contrast, the present study applied additional posterior-to-anterior manual pressure intraoperatively and synchronously with balloon inflation during PKP. Both groups underwent standard prone positioning; therefore, our comparison primarily reflects the incremental effect of the additional manual maneuver. The distinguishing feature of this technique is the timing of manual reduction and its coordination with balloon inflation. These studies underscore the potential advantages of postural reduction in enhancing vertebral height restoration. Jiang et al. (30) combined postural reduction with bilateral pedicle PKP for treating Kümmell's disease, achieving satisfactory pain relief and partial restoration of vertebral height, alongside kyphotic deformity correction. These findings suggest that postural reduction not only offers short-term benefits but may also yield positive effects in chronic fracture patients. These studies indicate that postural reduction plays a key role in improving vertebral height and kyphotic deformity correction, which inspired our own investigation. However, while previous studies primarily focused on postural reduction as a preoperative adjunct, our study evaluates the combined effect of postural reduction during PKP surgery itself, particularly examining whether manual postural reduction during balloon expansion enhances vertebral height restoration, kyphotic deformity correction, and pain relief during acute OVCF treatment.

In this study, we retrospectively compared the clinical and radiographic outcomes of PKP combined with postural reduction versus PKP alone in the treatment of acute thoracolumbar OVCF. Both groups demonstrated significant improvements in clinical function and radiographic outcomes, and our results indicate that PKP combined with postural reduction offers superior clinical benefits, particularly in improving ODI and LL at the one-year follow-up, compared to PKP alone. These findings are consistent with previous studies that highlight the advantages of postural reduction in improving functional outcomes and radiographic parameters. The mechanisms by which PKP alleviates pain are generally explained as follows: (1) Spinal Stability Enhancement: PKP restores vertebral stability and reduces external stress on the fracture site, thus preventing subsequent fractures. (2) Reduction in Nerve and Muscle Tension: PKP alleviates pain by reducing the strain on surrounding nerve and muscle tissues, which is especially effective in relieving lower back pain. (3) Pain Relief through Vertebral Restoration: The restoration of vertebral height during PKP reduces the load on surrounding structures, contributing to pain relief.

Both treatment groups experienced substantial postoperative improvements in pain and functional disability. However, no statistically significant between-group difference in VAS score was detected at any follow-up time point. This finding suggests that the addition of intraoperative postural reduction did not provide a detectable additional analgesic effect beyond that achieved with PKP alone. One possible explanation is that vertebral stabilization and reduction of fracture-site micromotion following cement augmentation may already provide substantial pain relief in both groups, leaving limited scope for an additional reduction in pain intensity through postural reduction. In contrast, Group A had a statistically lower ODI score at the 1-year follow-up. The ODI evaluates multiple dimensions of pain-related disability and daily activity, whereas the VAS primarily reflects pain intensity. Therefore, an isolated difference in ODI without a corresponding VAS difference could theoretically reflect small differences in mobility, posture, or performance of daily activities rather than additional pain relief. However, this interpretation remains speculative because these individual functional domains were not analyzed separately.

Importantly, the absolute between-group difference in the 1-year ODI score was only 2.84 points, which did not reach the commonly reported MCID of 10 points or the 12.8-point threshold reported in lumbar spine surgery populations. Both groups nevertheless demonstrated clinically meaningful within-group improvement: the ODI score decreased by 36.84 points in Group A and by 35.02 points in Group B. Thus, both treatments provided substantial functional improvement, whereas the additional functional benefit attributable to intraoperative postural reduction appears small and its clinical relevance remains uncertain. At the 1-year follow-up, Group A maintained a 4.11-percentage-point higher AVH percentage than Group B. This finding suggests that intraoperative postural reduction may contribute to greater preservation of vertebral height after PKP. Theoretically, better maintenance of anterior vertebral height may reduce vertebral wedging and local kyphotic deformation, thereby limiting abnormal loading of the anterior spinal column.

Intraoperatively, postural reduction involves positioning the patient prone and applying gentle manual pressure to extend the injured vertebra while simultaneously inflating the balloon. This action creates a ligamentotaxis effect, stretching the anterior column and longitudinal ligaments, thereby aiding in the restoration of vertebral height and reducing the kyphotic angle (31). When performed under appropriate conditions, manual postural reduction during PKP facilitates further vertebral height restoration and kyphosis correction, contributing to overall better outcomes in sagittal alignment. However, it is important to note that postural reduction may not be suitable for all patients. For instance, in cases of burst fractures associated with posterior longitudinal ligament injury, preoperative postural reduction may exacerbate the fracture or cause additional spinal cord injury. Therefore, careful preoperative imaging assessment is necessary to determine the suitability of postural reduction for each individual case.

OVCF often lead to sagittal imbalance in the spine, characterized by loss of anterior vertebral height, worsening kyphotic deformities, and anterior displacement of the spinal center of gravity (32–34). This imbalance results in increased mechanical stress on the spine and surrounding soft tissues, causing persistent back pain and potentially leading to neurological deficits (35). In our study, Group A demonstrated significantly better improvements in sagittal alignment, particularly with regard to lumbar lordosis (LL), at the final follow-up, which suggests that PKP combined with postural reduction is more effective at restoring sagittal balance than PKP alone. This may be due to the additional traction forces applied during postural reduction, which enhance the effects of PKP on vertebral height restoration and kyphosis correction, ultimately leading to more stable spinal alignment (36). No statistically significant between-group difference in VAS scores was observed at any follow-up time point. At 1 year, Group A had a lower ODI score than Group B (18.71 ± 4.72 vs. 21.55 ± 4.92) however, the 2.84-point difference did not reach the conventional MCID threshold. This apparent dissociation between pain and functional outcomes may reflect the different constructs assessed by VAS and ODI. Because both groups underwent PKP, cement-mediated stabilization of the fractured vertebra and reduction of fracture-site micromotion may have accounted for most of the pain relief in both groups. The relatively low postoperative VAS scores may also have produced a potential floor effect, leaving limited room to detect an additional analgesic benefit from positional reduction.

In contrast, ODI reflects broader pain-related disability, including limitations in standing, walking, lifting, and other daily activities. The greater preservation of AVH and regional lumbar alignment in Group A might theoretically improve load distribution, trunk mechanics, postural endurance, and mobility, thereby producing a small difference in functional outcome without a corresponding reduction in perceived pain intensity. Nevertheless, this explanation remains hypothetical. We did not analyze patient-level associations between changes in AVH or LL and changes in ODI. Therefore, the present findings should not be interpreted as evidence that sagittal balance restoration caused the observed functional difference. Instead, they indicate comparable pain relief between the two groups, accompanied by greater regional radiographic correction and a small ODI difference.

The incidence of bone cement leakage was numerically higher in Group A than in Group B (9.68% vs. 4.55%), although the difference was not statistically significant. This trend warrants attention because intraoperative manual reduction may theoretically alter the morphology of the fractured vertebra. External pressure applied during balloon inflation could increase fracture mobility, enlarge pre-existing fracture lines or intravertebral clefts, or create communication between the balloon-created cavity and a cortical or endplate defect. These changes could potentially provide a pathway for cement extravasation during subsequent injection. However, this proposed mechanism remains speculative. Cement leakage is multifactorial and may also be influenced by cortical disruption, fracture severity, cement viscosity, injection timing, cement volume, and venous anatomy. In the present study, the cement volumes were comparable between the two groups, and the small number of leakage events precluded an adjusted risk analysis. Therefore, our findings do not demonstrate that manual reduction increases the risk of cement leakage. Nevertheless, careful preoperative evaluation of cortical defects, gentle and fluoroscopically monitored reduction, controlled low-pressure cement injection, and prospective documentation of leakage patterns are warranted in future studies. The combined technique may also involve a learning curve. In the present study, manual reduction was performed by the same experienced surgeon using gradual posterior-to-anterior pressure under intermittent C-arm fluoroscopy and neuromonitoring, which reduced interoperator variability. However, the applied force was not quantitatively measured, and the learning curve of the technique was not formally evaluated. Improper hand positioning, excessive force, or pursuit of overcorrection may increase the risk of additional vertebral injury or neurological complications, particularly in patients with severe osteoporosis, posterior wall disruption, spinal canal compromise, burst fractures, or suspected posterior longitudinal ligament injury. Accordingly, careful preoperative CT and MRI assessment, strict patient selection, gradual force application, and continuous radiographic and neurological monitoring are essential. Future studies should establish standardized force thresholds, procedural stopping criteria, and structured training protocols to improve the reproducibility and safety of this technique. Meanwhile, vertebral puncture and cement injection should be performed under strict C-arm fluoroscopic guidance. Careful control of injection pressure, timing, and cement volume is essential to minimize the risk of cement leakage and prevent adverse outcomes.

This study has several limitations. First, its retrospective, single-centre design and non-random treatment allocation introduce potential selection bias, confounding by indication, and unmeasured confounding, precluding causal inference. The small sample (75 patients; 31 vs. 44) and few complications limited statistical power, while unadjusted multiple comparisons increased the risk of type I error. Multivariable regression was not performed because of the limited sample size and number of events. Second, outcome assessment was not blinded, and all radiographic measurements were performed by a single observer, creating potential detection and measurement bias. Third, although manual reduction was performed by the same experienced surgeon, the applied pressure was neither standardised nor quantified, limiting reproducibility. Excessive force may theoretically propagate fractures or injure surrounding structures in osteoporotic patients. Fourth, the mean follow-up of 17.45 months may be insufficient to evaluate the durability of radiographic correction and delayed complications, including adjacent vertebral fractures and vertebral recollapse. The single-centre setting and exclusion of patients with neurological symptoms or unstable fractures further limit generalisability. Finally, full-length standing radiographs and global spinopelvic parameters, including PI, PT, SVA, TPA, and PI–LL mismatch, were not assessed. The relationships between changes in AVH or LL and ODI were also not analysed; therefore, whether the observed LL difference affected global sagittal balance or functional outcomes remains uncertain. Future multicentre prospective studies with larger samples, blinded assessment, standardised reduction protocols, comprehensive sagittal evaluation, and longer follow-up are warranted.

In this study, we selected patients with OVCF occurring within two weeks to minimize potential biases. Future studies should focus on sagittal spinal alignment in such patients, as this plays a significant role in correcting overall spinal deformities.

## Conclusion

Both PKP and PKP combined with postural reduction effectively relieve pain and improve functional outcomes in patients with acute OVCF. However, PKP combined with postural reduction demonstrates superior results in restoring AVH, correcting kyphotic deformities, and preserving regional lumbar alignment compared to PKP alone. However, no additional analgesic benefit was detected, and the small between-group difference in the 1-year ODI score did not reach the conventional MCID threshold. Therefore, the clinical relevance of the observed functional difference remains uncertain. PKP combined with postural reduction is a promising treatment option for suitable patients with acute OVCF and is recommended for broader clinical application. It may not be suitable for unstable burst fractures or cases with posterior wall disruption, spinal canal compromise, neurological deficits, or suspected posterior longitudinal ligament injury. Careful imaging assessment, gentle force application, and appropriate intraoperative monitoring are essential to reduce the risks of cement leakage, overcorrection, and further vertebral injury.

## Data Availability

The original contributions presented in the study are included in the article/Supplementary Material, further inquiries can be directed to the corresponding authors.

## References

[1] Roux C Wyman A Hooven FH Gehlbach SH Adachi JD Chapurlat RD. Burden of non-hip, non-vertebral fractures on quality of life in postmenopausal women: the global longitudinal study of osteoporosis in women (GLOW). Osteoporos Int. (2012) 23(12):2863–71. 10.1007/s00198-012-1935-8 22398855 PMC4881739

[2] Wilhelm K. Vertebroplasty–state of the art. Radiologe. (2015) 55(10):847–52. 10.1007/s00117-015-0013-6 26358361

[3] Theodorou DJ Theodorou SJ Duncan TD Garfin SR Wong WH. Percutaneous balloon kyphoplasty for the correction of spinal deformity in painful vertebral body compression fractures. Clin Imaging. (2002) 26(1):1–5. 10.1016/S0899-7071(01)00350-3 11814744

[4] Al Taha K Lauper N Bauer DE Tsoupras A Tessitore E Biver E. Multidisciplinary and coordinated management of osteoporotic vertebral compression fractures: current state of the art. J Clin Med. (2024) 13(4):930. 10.3390/jcm13040930 38398244 PMC10889683

[5] Anselmetti GC Bernard J Blattert T Court C Fagan D Fransen H. Criteria for the appropriate treatment of osteoporotic vertebral compression fractures. Pain Physician. (2013) 16(5):E519–30. 10.36076/ppj.2013/16/E519 24077202

[6] Harrop JS Prpa B Reinhardt MK Lieberman I. Primary and secondary osteoporosis' Incidence of subsequent vertebral compression fractures after kyphoplasty. Spine (Phila Pa 1976). (2004) 29(19):2120–5. 10.1097/01.brs.0000141176.63158.8e 15454702

[7] Martikos K Greggi T Faldini C Vommaro F Scarale A. Osteoporotic thoracolumbar compression fractures: long-term retrospective comparison between vertebroplasty and conservative treatment. Eur Spine J. (2018) 27(Suppl 2):244–7. 10.1007/s00586-018-5605-1 29675674

[8] Taylor RS Fritzell P Taylor RJ. Balloon kyphoplasty in the management of vertebral compression fractures: an updated systematic review and meta-analysis. Eur Spine J. (2007) 16(8):1085–100. 10.1007/s00586-007-0308-z 17277923 PMC2200787

[9] Yang CC Chien JT Tsai TY Yeh KT Lee RP Wu WT. Earlier vertebroplasty for osteoporotic thoracolumbar compression fracture may minimize the subsequent development of adjacent fractures: a retrospective study. Pain Physician. (2018) 21(5):E483–e91. 10.36076/ppj.2018.5.E483 30282396

[10] Yang D Zhang Y Ma X Huo L Li L Gao Y. Resources utilisation and economic burden of percutaneous vertebroplasty or percutaneous kyphoplasty for treatment of osteoporotic vertebral compression fractures in China: a retrospective claim database study. BMC Musculoskelet Disord. (2020) 21(1):255. 10.1186/s12891-020-03279-1 32303207 PMC7165412

[11] Zhang SM Zhang ZJ Liu YZ Zhang LT Li X. Treatment of senile osteoporotic vertebral compression fractures with percutaneous kyphoplasty. Zhongguo Gu Shang. (2011) 24(7):570–3. 21870396

[12] Garfin SR Yuan HA Reiley MA. New technologies in spine: kyphoplasty and vertebroplasty for the treatment of painful osteoporotic compression fractures. Spine (Phila Pa 1976). (2001) 26(14):1511–5. 10.1097/00007632-200107150-00002 11462078

[13] Lieberman IH Dudeney S Reinhardt MK Bell G. Initial outcome and efficacy of “kyphoplasty” in the treatment of painful osteoporotic vertebral compression fractures. Spine. ((Phila Pa 1976). 2001) 26(14):1631–8. 10.1097/00007632-200107150-00026 11464159

[14] Savage JW Schroeder GD Anderson PA. Vertebroplasty and kyphoplasty for the treatment of osteoporotic vertebral compression fractures. J Am Acad Orthop Surg. (2014) 22(10):653–64. 10.5435/JAAOS-22-10-653 25281260

[15] Kim K-H Kuh S-U Chin D-K Jin B-H Kim K-S Yoon Y-S. Kyphoplasty versus vertebroplasty: restoration of vertebral body height and correction of kyphotic deformity with special attention to the shape of the fractured vertebrae. J Spinal Disord Tech. (2012) 25(6):338–44. 10.1097/BSD.0b013e318224a6e6 21705918

[16] Liu JT Liao WJ Tan WC Lee JK Liu CH Chen YH. Balloon kyphoplasty versus vertebroplasty for treatment of osteoporotic vertebral compression fracture: a prospective, comparative, and randomized clinical study. Osteoporos Int. (2010) 21(2):359–64. 10.1007/s00198-009-0952-8 19513578

[17] Filippiadis DK Marcia S Masala S Deschamps F Kelekis A. Percutaneous vertebroplasty and kyphoplasty: current status, new developments and old controversies. Cardiovasc Intervent Radiol. (2017) 40(12):1815–23. 10.1007/s00270-017-1779-x 28856402

[18] Gu CN Brinjikji W Evans AJ Murad MH Kallmes DF. Outcomes of vertebroplasty compared with kyphoplasty: a systematic review and meta-analysis. J Neurointerv Surg. (2016) 8(6):636–42. 10.1136/neurintsurg-2015-011714 25964376

[19] Gonschorek O Hauck S Weiß T Bühren V. Percutaneous vertebral augmentation in fragility fractures-indications and limitations. Eur J Trauma Emerg Surg. (2017) 43(1):9–17. 10.1007/s00068-016-0753-7 28101655

[20] Sanli I Van Kuijk SMJ De Bie RA Van Rhijn LW Willems PC. Percutaneous cement augmentation in the treatment of osteoporotic vertebral fractures (OVFs) in the elderly: a systematic review. Eur Spine J. (2020) 29(7):1553–72. 10.1007/s00586-020-06391-x 32240375

[21] Wang G Yang H Chen K. Osteoporotic vertebral compression fractures with an intravertebral cleft treated by percutaneous balloon kyphoplasty. J Bone Joint Surg Br. (2010) 92(11):1553–7. 10.1302/0301-620X.92B11.24671 21037351

[22] Cerny J Soukup J Petrosian K Loukotova L Novotny T. Efficacy and complication rates of percutaneous vertebroplasty and kyphoplasty in the treatment of vertebral compression fractures: a retrospective analysis of 280 patients. J Clin Med. (2024) 13(5):1495. 10.3390/jcm13051495 38592338 PMC10933847

[23] Wang H Zhang L Du H Wu X. Efficacy and safety evaluation of bilateral pedicle approach combined with positional reduction for the treatment of osteoporotic thoracolumbar burst fractures. Altern Ther Health Med. (2023) 29(6):176–81. 37235499

[24] Jeon CH Lee YS Youn SJ Lee HD Chung NS. Factors affecting postural reduction in posterior surgery for thoracolumbar burst fracture. J Spinal Disord Tech. (2015) 28(4):E225–30. 10.1097/BSD.0000000000000208 25353208

[25] Pourabbas Tahvildari B Safari R Pouralimohammadi MA. Analysis of the effect and prognostic factors associated with postural and instrumental reduction in thoracolumbar burst fractures. J Biomed Phys Eng. (2021) 11(3):337–44. 10.31661/jbpe.v0i0.2012-1245 34189122 PMC8236101

[26] Hu Y Liu S Yang R Wang H. Auxiliary manual relocation and reduction for patients with thoracolumbar compression fracture- efficacy, safety and technical notes. Asian J Surg. (2023) 46(11):5222–3. 10.1016/j.asjsur.2023.07.004 37481361

[27] Haarbo J Gotfredsen A Hassager C Christiansen C. Validation of body composition by dual energy x-ray absorptiometry (DEXA). Clin Physiol. (1991) 11(4):331–41. 10.1111/j.1475-097X.1991.tb00662.x 1914437

[28] Chin DK Kim YS Cho YE Shin JJ. Efficacy of postural reduction in osteoporotic vertebral compression fractures followed by percutaneous vertebroplasty. Neurosurgery. (2006) 58(4):695–700. 10.1227/01.NEU.0000204313.36531.79 16575333

[29] Yang JJ Koo KH Kim K Park S. Efficacy of postural reduction of vertebral compression fracture with extension lateral radiograph before vertebroplasty. World Neurosurg. (2020) 143:e430–e41. 10.1016/j.wneu.2020.07.188 32750516

[30] Jiang J Gu FL Li ZW Zhou Y. The clinical efficacy and experience of bipedicular percutaneous vertebroplasty combined with postural reduction in the treatment of Kümmell's Disease. BMC Musculoskelet Disord. (2020) 21(1):82. 10.1186/s12891-020-3113-z 32033554 PMC7007684

[31] Xu Y Zhou X Yu C Cheng M Dong Q Qian Z. Effectiveness of postural and instrumental reduction in the treatment of thoracolumbar vertebra fracture. Int Orthop. (2008) 32(3):361–5. 10.1007/s00264-007-0338-5 17333182 PMC2323410

[32] Yokoyama K Ikeda N Tanaka H Ito Y Sugie A Yamada M. Changes in spinal sagittal balance after a new osteoporotic vertebral compression fracture. Osteoporos Int. (2024) 35(4):645–51. 10.1007/s00198-023-06976-4 38108858

[33] Sutipornpalangkul W Zambrana L Gianakos A Lane JM. Comparison of sagittal balance between radiofrequency targeted vertebral augmentation and balloon kyphoplasty in treatment of vertebral compression fracture: a retrospective study. J Med Assoc Thai. (2016) 99(9):1025–32. 29927208

[34] Yokoyama K Kawanishi M Yamada M Tanaka H Ito Y Kawabata S. Postoperative change in sagittal balance after kyphoplasty for the treatment of osteoporotic vertebral compression fracture. Eur Spine J. (2015) 24(4):744–9. 10.1007/s00586-014-3678-z 25404372

[35] Najjar E Pasku D Mardashti A Meshneb M Komaitis S Salem KM. The influence of osteoporotic vertebral fractures on global sagittal alignment in elderly patients: a systematic review and meta-analysis. Eur Spine J. (2023) 32(7):2580–7. 10.1007/s00586-023-07780-8 37222801

[36] Cao Z Wang G Hui W Liu B Liu Z Sun J. Percutaneous kyphoplasty for osteoporotic vertebral compression fractures improves spino-pelvic alignment and global sagittal balance maximally in the thoracolumbar region. PLoS One. (2020) 15(1):e0228341. 10.1371/journal.pone.0228341 31999783 PMC6992186

